# Genetic aberrations of c*-myc* and *CCND1* in the development of invasive bladder cancer

**DOI:** 10.1038/sj.bjc.6600531

**Published:** 2002-09-04

**Authors:** A D Watters, Z Latif, A Forsyth, I Dunn, M A Underwood, K M Grigor, J M S Bartlett

**Affiliations:** University Department of Surgery, Level II, Queen Elizabeth Building, Glasgow Royal Infirmary, Glasgow G31 2ER, UK; Department of Surgery, Southern General Hospital, Glasgow, UK; University Department of Pathology, Western General Hospital, Edinburgh EH4 2XU, UK

**Keywords:** transitional cell carcinoma, oncogenes, prognosis, c-*myc*, *CCND1*

## Abstract

Detrusor muscle invasive transitional cell carcinoma is associated with poor prognosis and is responsible for the majority of bladder cancer related deaths. Amplifications of c*-myc* and *CCND1* are associated with detrusor-muscle-invasive transitional cell carcinoma, however, their precise role in driving disease progression is unclear. Fluorescence *in situ* hybridisation on archival tissue from 16 patients with primary diagnosis of ⩾pT2 transitional cell carcinoma and 15 cases with primary pTa/pT1 disease subsequently progressing to detrusor-muscle-invasion was performed, in the latter group both pre and post muscle invasive events were studied. No patients presenting with ⩾pT2 had amplification of c*-myc*, two out of 16 (12.5%) had *CCND1* amplification. Of patients who developed ⩾pT2, two out of 15 (13.3%) had amplification of c*-myc*, both in ⩾pT2, five out of 15 (33.3%) had *CCND1* amplification, two in pTa/pT1 tumours, three in ⩾pT2 transitional cell carcinomas. In total, two out of 31 (6.5%) of patients' ⩾pT2 TCCs were amplified for c-*myc* and six out of 31 (19%) were amplified for *CCND1*. Eighty-seven per cent (40 out of 46) of tumours were polysomic for chromosome 8 and 80% (37 out of 46) were polysomic for chromosome 11 and this reflected the high copy numbers of c-*myc* and *CCND1* observed. In almost all cases an increase in c-*myc/CCND1* copy number occurred prior to invasion and persisted in advanced disease. Amplification of *CCND1* or alterations in c-*myc/CCND1* early in bladder cancer may have clinical relevance in promoting and predicting progression to detrusor-muscle-invasive transitional cell carcinoma.

*British Journal of Cancer* (2002) **87**, 654–658. doi:10.1038/sj.bjc.6600531 www.bjcancer.com

© 2002 Cancer Research UK

## 

Muscle invasive transitional cell carcinoma (TCC) of the urinary bladder (characterised by invasion to the detrusor-muscle wall of the bladder or beyond) has a poor prognosis. Invasive TCC is routinely treated with radiotherapy or surgery but, despite treatment, up to 50% of patients develop metastases and die within 5 years (reviewed by van der Meijden, 1998). A large number of studies have attempted to identify which molecular markers may be driving progression, ultimately to develop targeted therapies (reviewed by Unwin *et al*, 1999; Reznikoff *et al*, 2000; Witjes *et al*, 2000). Cell cycle regulators such as p53 and Rb have been implicated in bladder cancer progression (reviewed by Knowles, 1999).

Previous research within this laboratory demonstrated that polysomy of chromosomes 9, 7 and 17 are associated with recurrence (Bartlett *et al*, 1998, Watters *et al*, 2000), but that these genetic events are not linked with polyploidy (Bartlett *et al*, 1999). A small study within this laboratory investigating the drug metabolising enzyme NAT2 (the gene is located at chromosome 8p22) in TCC recurrence or progression suggested that polysomy 8 was associated with disease progression (Watters *et al*, 2001). Further research showed an association with polysomy of one or eight in preinvasive TCCs associated with subsequent invasion (Watters *et al*, 2002).

Gene amplification has been postulated as a mechanism driving progression in many carcinomas. c-*myc*, located at 8q24, encodes for a nuclear transcription factor that regulates cell proliferation and differentiation (Visscher *et al*, 1997). Gene amplification or increased copy number of c-*myc* has been reported in breast (Visscher *et al*, 1997), head and neck and ovarian cancer (Sauter *et al*, 1995). There has been a suggestion that gain of chromosome 8q is involved in progression in bladder cancer (Sauter *et al*, 1995). 8q24 gains have been associated with advanced stage and grade bladder cancer (Wagner *et al*, 1997). Low rates of amplification (15%) at the 11q13 region have been reported in bladder cancer (reviewed by Schuuring, 1995). *CCND1* maps to 11q13 (Bringuier *et al*, 1996), involved in the regulation of the G1/S phase transition of the cell cycle (Bringuier *et al*, 1996).

Following on from previous studies within this laboratory (Bartlett *et al*, 1998, 1999; Watters *et al*, 2000, 2001) which associated specific chromosomal aberrations with recurrence or progression, we hypothesised that alteration of key cell cycle modulators c-*myc* and *CCND1* via gene alterations drives progression of superficial and locally invasive TCC (pTa/pT1). This study determined chromosome and gene copy number by FISH in a cohort of patients with either detrusor muscle invasive TCC at presentation or who developed detrusor muscle invasive TCC following a presentation with non-muscle invasive (pTa/pT1) TCC.

## MATERIALS AND METHODS

### Patients

Sixteen patients with ⩾pT2 disease and seven with pTa and eight with pT1 disease that progressed to ⩾pT2 were identified from a bladder cancer database in the Department of Surgery, Glasgow Royal Infirmary. Patients were categorised into two groups: PP (progressed at presentation; patients with ⩾pT2 at initial diagnosis) and RP (recurrent and progressive; patients with pTa/pT1 disease at initial diagnosis who subsequently recurred and progressed with their disease). In order to attempt to understand the genetic events underlying progression from pTa or pT1 TCC, the pre and post muscle-events were studied in the RP group; the presenting lesion in the PP group. This approach was based on previous research within this laboratory that demonstrated that there was a considerable increase in genetic aberrations from patients with a primary pTa or pT1 TCC that progressed to detrusor-muscle-invasion when compared to those with non-invasive recurrences (Watters *et al*, 2002).

A total of 46 tumours were studied. Five micron formalin fixed paraffin processed tissue sections of each tumour were cut onto sialinised slides and baked at 56°C overnight. All TCCs analysed had a representative section stained with haematoxylin and eosin and were restaged and regraded by a specialist urological pathologist (KMG) following current UICC guidelines (Sobin and Wittekind, 1995).

### Fluorescence *in situ* hybridisation (FISH)

To determine gene and chromosome copy numbers, levels of gene amplification or deletion *in situ*, FISH, using locus specific DNA probes for c-*myc*, *CCND1* and chromosome enumeration probes for eight and 11 in a dual hybridisation reaction, was applied to five micron tissue sections of tumour. Included in each run was bladder tissue with normal copy number for all the probes used.

The FISH methodology was followed as outlined: Tissue sections were dewaxed and rehydrated, then subjected to pretreatments 0.2 N HCl for 20 min at room temperature, 8% sodium thiosulphate at 80°C for 30 min and 0.5% pepsin in 0.2 N HCl for 26 min at 37°C. Tissue sections were post-fixed in 10% neutral buffered formalin at room temperature for 10 min before dehydration in ascending grades of alcohol and air drying. These steps were carried out on a VP2000 robotic pretreatment slide processor (Vysis, UK, Ltd).

The tissue sections were assessed for extent of digestion as previously outlined (Watters *et al*, 2000). Tissue sections were denatured in 70% formamide, 2×SSC pH 7–8 at 72°C for 5 min on the Omnislide hybridisation module (Hybaid, UK Ltd), dehydrated and air-dried. Two separate experiments were carried out on adjacent sections, with probes for the pericentromere of chromosome 8, labelled with SpectrumGreen™ and a SpectrumOrange™ labelled probe for c-*myc* (Vysis, UK). The alternative probes set was probes for the pericentromere of chromosome 11, labelled with SpectrumGreen™ and a SpectrumOrange™ labelled probe for *CCND1* (Vysis, UK). Both combinations were applied as a double label. For each section, 1 μl of each probe was added to 7 μl hybridisation mix (50% formamide, 2×SSC, 10% dextran sulphate) and 1 μl deionised water and denatured in a waterbath at 72°C for 5 min, applied to the tissue sections and hybridised overnight at 37°C (Vysis, Inc).

Following posthybridisation washes in 0.4×SSC, 0.3% Nonidet 30, pH 7 at 72°C for 2 min, the tissue sections were mounted in 0.25 μg ml^−1^ DAPI in antifade (Vectashield, UK) and viewed with a Leica DMLB microscope. A triple band pass filter block spanning the excitation and emission wavelengths of the SpectrumGreen™ and SpectrumOrange™ fluors (Vysis, UK) and DAPI was used in the analysis of the hybridisation. Image capture was achieved using a digital camera and software (Leica DC200, Leica UK).

### Scoring criteria

Serially sectioned haematoxylin and eosin stained tissue sections were first examined microscopically to localise areas of TCC. FISH stained sections were then scanned at ×400 magnification to localise the TCC areas identified. Three distinct areas were identified and signals per nucleus in 20 nuclei per area for chromosome 8 and c-*myc* and chromosome 11 and *CCND1* respectively were counted at ×1000 magnification and the results recorded manually. Average chromosome and gene copy number per area was calculated by totalling the number of signals counted and dividing this figure by the number of nuclei assessed. Mean copy number per section was calculated by combining the results for the three areas. Where chromosomal heterogeneity was noted the most abnormal score was used in the final analysis. Control sections from normal bladder tissue obtained from post mortem normal bladder were included in the analysis. Values for disomy were derived from the analysis of these tissue sections as previously reported (Bartlett *et al*, 1998; Watters *et al*, 2000).

The ratio of gene to chromosome copy number was determined by dividing the number of signals for c-*myc* or *CCND1* by the number of signals for chromosome 8 or 11 respectively. Gene amplification was defined as a ratio greater than two, based on values determined by Vysis Inc and incorporated in their guidelines for using the PathVysion™ system to determine amplification levels of the *HER2/neu* gene in breast cancer in a diagnostic setting (Bartlett *et al*, 2001). This was based on the normal mean copy number for the gene as being less than four signals per nucleus. In some cases with high amplification ratios (>5 copies per chromosome), clusters of gene signals, analogous to homogeneous staining regions, were observed. In these cases it was not possible to accurately assess the true gene copy number, but by focusing through the cluster a reasonable estimate was achieved. In none of these cases did the presence of a cluster of signals alter the final result.

### Statistics

Crosstabs (SPSS for windows release 9.0) was used to compare similarity of copy number between chromosomes 8 and 11 and c-*myc* and *CCND1*.

## RESULTS

### Patients

The average age of the PP group was 70.9 years, (range 50 to 89 years) and the RP group 68.9 years (range 43 to 84 years). The M : F ratio was 13 : 3 in the PP group, 12 : 3 in the RP group. Mean follow-up for the PP group was 42 months (range 6–91) and for the RP group 39 months (range 3–117). In the PP group, 13 patients were treated with radiotherapy, one died before radiotherapy commenced, one refused radiotherapy and one had a cystectomy. In the RP group, six had radiotherapy after the muscle-invasive event, five had a cystectomy, two had no treatment. One patient had palliative radiotherapy early in his disease history, subsequently recurred with pT1 and pTa TCCs and eventually, 13 years later, succumbed to his disease, and one patient had radiotherapy before his muscle-invasive TCC. Of the PP group, 11 patients died from their disease, range 6 to 59 months following diagnosis. The four patients still alive had no evidence of local or metastatic disease at last clinic appointment. Of the RP group 9 patients died of their disease, range 3–15 months following diagnosis of invasive disease. Of the six patients still alive, two had no evidence of local or metastatic disease at last clinic appointment, two had metastatic disease, and there were no records following diagnosis with invasive TCC for the remaining two.

### FISH

All hybridisations were successful apart from that of one PP with the probes for c-*myc* and chromosome 8. The normal range for copy number of each DNA probe is detailed in Table 1

**Table 1:**
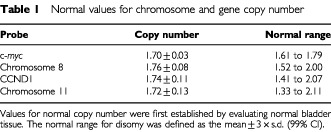
Normal values for chromosome and gene copy number

, and an example of a TCC with disomy of c-*myc* and chromosome 8 is shown in Figure 1A

**Figure 1:**
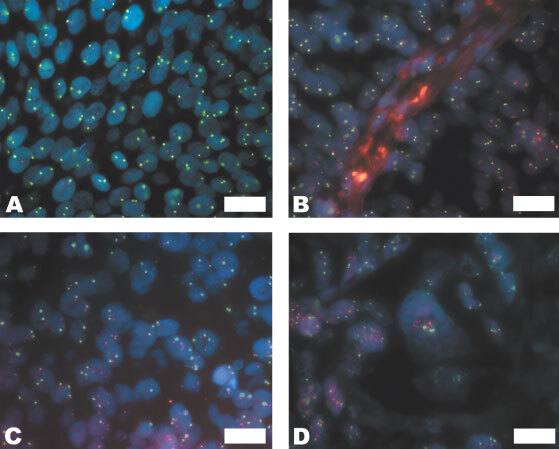
FISH size bar on all photomicrographs is 20 microns. Chromosome copy depicted in green, gene copy in red, nuclei stained with DAPI, fluorescing blue. (**A**) Disomy of c-*myc* and chromosome 8, PP pT2 G3. (**B**) Polysomy of c-*myc* and chromosome 8, RP pT1cG3. (**C**) Polysomy of *CCND1* and chromosome 11, RP, pT2G3. (**D**) Amplification of *CCND1*, RP, pT2G3.

.

### c*-myc* and chromosome 8

Fourteen out of 15 (93%) PP tumours had increased copy number of c-*myc* and chromosome 8, between 2.15 and 6.35 for c-*myc* and between 2.03 and 7.73 for chromosome 8 (Figure 1B). None of the tumours exhibited gene amplification (Table 2

**Table 2:**
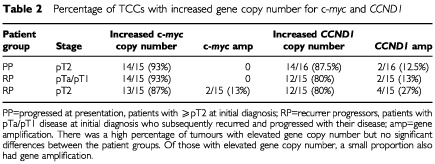
Percentage of TCCs with increased gene copy number for c-*myc* and *CCND1*

).

Twenty-seven out of 30 (90%) RP tumours had increased copy number for c-*myc* of between 2.03 and 5.53. Of these 14 out of 15 (93%) were abnormal in the pTa and pT1 TCCs, the TCC was pTa and 13 out of 15 (87%) were abnormal in the ⩾pT2 TCC (Table 2). Twenty-seven out of 30 (90%) RP tumours had polysomy 8, (range 2.05 to 4.89). Of these 13 out of 15 (87%) were polysomic in the pTa or pT1 TCCs , both normal TCCs were pTa and 14 out of 15 (93%) were polysomic in the pT2+ TCC. Two out of 30 (6.7%) tumours had gene amplification, and both were ⩾pT2.

There was a close correlation between c-*myc* and chromosome 8 copy number, *P*=0.001.

### *CCND1* and chromosome 11

Fourteen out of 16 (87.5%) PP tumours had an increase of *CCND1*, copy number between 2.70 and 13.18 (Table 2) and 12 out of 16 (75%) had polysomy 11, copy number between 2.70 and 5.88 (Figure 1C). Of these, two (12.5%) had amplification of *CCND1* (Table 2, Figure 1D).

Twenty-four out of 30 (80%) RP tumours had an increase in *CCND1* (range 2.11 to 9.00, Table 2). Of these 12 out of 15 (80%) were abnormal in the pTa or pT1 TCCs; two of the normal tumours were pTa, one was pT1, and 12 out of 15 (80%) were abnormal in the pT2+ TCC. Twenty-three out of 30 (77%) RP tumours were polysomic for chromosome 11, copy number between 2.13 and 4.57. Of these 11 out of 15 (73%) were polysomic in the pTa or pT1 TCC; three of the normal TCCs were pTa, one was pT1, and 12 out of 15 (80%) were polysomic in the pT2+ TCC. Six out of 30 (20%) tumours had amplification of *CCND1*, four of which were pT2 or above, the remaining two were pT1G3 and pTaG3. Of these one patient had amplification in their pre and post muscle invasive tumours. There was a close correlation with *CCND1* and chromosome 11 copy number, *P*<0.001.

In total six out of 31 patients (19%) had amplification of *CCND1* in their detrusor muscle invasive tumours, compared to two out of 31 (6.5%) patients with amplification of c*-myc*, N/S. Both patients with amplification of c-*myc* had amplification of *CCND1*.

Data from the results of the assessment of gene copy number in the non-muscle invasive (pTa or pT1) TCCs is depicted in Table 3

**Table 3:**
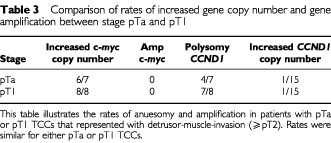
Comparison of rates of increased gene copy number and gene amplification between stage pTa and pT1

. This shows similar rates of increased copy number in pTa or pT1 tumours.

## DISCUSSION

The observation that amplification of oncogenes predominates in later stages of cancer progression (Ried *et al*, 1999; Reznikoff *et al*, 2000) and that the majority of genetic alterations in TCC occur prior to progression (Tsao *et al*, 2000) implies that genetic instability of this type may influence progression. The aim of this study was to determine if changes of the oncogenes c-*myc* and *CCND1* in preinvasive tumours drive later progression. Abnormal regulation of c-*myc* can result in phenotypic transformation, aberrant cell cycle control and genomic instability. Disruption of *CCND1*, that encodes for cyclin D1, involved in the regulation of the G1/S transition of the cell cycle (Bringuier *et al*, 1996), may form the basis of genetic instability (Reznikoff *et al*, 2000).

The small numbers of tumours exhibiting gene amplification of c-*myc* observed here is in accordance with previous studies (Sauter *et al*, 1995). However, rates of amplification of *CCND1* were 19%, marginally higher than a previous study of bladder cancer by Southern blotting which reported amplification rates of 11q13 of 11% (Bringuier *et al*, 1996). The candidate oncogenes in the region 11q13 in bladder cancer postulated by Bringuier *et al* (1996) are *CCND1* and *EMS1* (a gene involved in regulating the interactions between components in the adherens junctions, Schuuring (1995)).

Both the 8q24 (that contains c-*myc*) and 11q13 regions are frequently involved in translocations in lymphomas (Arber, 2000). Burkitt's lymphoma is usually associated with a translocation of c-*myc* and *CCND1* has been suggested as a marker for mantle cell lymphoma (Arber, 2000). Although translocations are not uncommon in bladder cancer, translocation as a sole cytogenetic event has not been described (Sandberg and Berger, 1994). However, the chromosomal regions that contain c-*myc* and *CCND1* are genetically unstable, which may be of significance in the development of muscle-invasive bladder cancer.

Previously, research from this laboratory has shown an observed increase in aneusomy from pTa to pT1 TCCs, but a lack of association with stage and recurrence or progression, suggesting that it is aneusomy and not pathological classification which is driving progression in pTa or pT1 tumours (Watters *et al*, 2001, 2002; Edwards *et al*, 2001). Furthermore, it is of interest to note that the rate of genetic abnormalities is similar when comparing the data from patients who progress from non-muscle-invasion and those who present with muscle invasion. In this study, and previously, we have shown that there are higher rates of aneusomy in patients who progress to muscle invasion compared to those who recur with pTa/pT1 disease (Watters *et al*, 2000, 2001, 2002). Muscle-invasive TCCs are characterised by a large number of cytogenetic alterations including overrepresentation of 8q and 11q13 (Tomovska *et al*, 2001). Thus this study would support the finding by Tsao *et al* (2000) that the majority of genetic alterations in TCC occur prior to progression. They showed that there was an increase of only 10% of genetic aberrations in metastases compared to primary cell lines from two patients reported in their study, and they suggest that most allelic losses occur before progression. It is widely recognised that genetic instability increases from precursor, or in the case of TCC, preinvasive lesions, to invasion (Ried *et al*, 1999). In our study we also observed that there was a very high rate of abnormalities in preinvasive lesions, regardless of stage.

A gain of chromosome 8q may contribute to bladder cancer progression (Sauter *et al*, 1995). The gain of a large fragment of 8q also known as an isochrome 8q has been observed in bladder cancer (Sauter *et al*, 1995). The stronger increase in 8q24 gains from pT1 to pT2–4 than from pTa to pT1 could suggest that 8q gains occur later in bladder cancer progression than 8p deletions (Wagner *et al*, 1997). In a study of chromosomal aberrations by comparative genomic hybridisation, there was a very small percentage of gains of 11q13 in pTa tumours. However, in pT1 tumours, loss of chromosome 11 was observed with the exception of 11q13 which was often gained or amplified (Simon *et al*, 1998). In this study, c-*myc* and *CCND1* may be dysfunctional due to the observed increased copy number, and it is possible that protein expression is increased as a consequence which may be a route to muscle-invasion.

When the results from this study were compared to a previous study in this laboratory that examined *HER2/neu* and chromosome 17 levels in TCC (unpublished data), very similar results were obtained i.e high levels of polysomy and much lower levels of amplification. This suggests that tumours that progress to pT2 and above, or present with that phenotype, have high levels of polysomy of many chromosomes, and that they may exhibit polyploidy. However, we have investigated, by the use of multiple FISH probes, the rate of polysomy in this cohort and have found that this cannot explain the findings in this study (Bartlett *et al*, 1999). Therefore we conclude that the alterations in these chromosomes represent events which are closely associated with disease progression and independent of polyploidisation. Ried *et al* (1999) noted that the number of chromosomal copy alterations increased significantly in the transition from precursor lesions to invasive carcinomas, reflecting an overall genetic instability which we have also observed here.

Further studies of gene alterations in non-invasive non progressive patient groups and investigations of protein expression are required to clarify the effects of genetic abnormalities of c-*myc* and *CCND1* on progression of TCC. Nevertheless, this study has added to the growing evidence that genetic alterations prior to detrusor muscle invasion delineate those cancers which will progress and by extension these changes are associated with genes which therefore drive progression.
